# Effects of Adding Blenderized Tube Feeds to Commercial Enteral Formulas on Gastrointestinal Symptoms in Adult Patients Receiving Enteral Nutrition

**DOI:** 10.3390/medicina62101839

**Published:** 2026-09-23

**Authors:** Athanasios Migdanis, Constantinos Giaginis, Ioanna Partsalaki, Vasiliki Bismpardi, Ioannis Migdanis, Sousana K. Papadopoulou, Maria Anna Polyzou Konsta, Agathi Pritsa, Odysseas Androutsos

**Affiliations:** 1 Nutrition and Dietetics Department, University General Hospital of Larisa, 41110 Larissa, Greece; 2 Lab of Clinical Nutrition and Dietetics, Nutrition and Dietetics Department, University of Thessaly, 42132 Trikala, Greece; oandroutsos@uth.gr; 3 Department of Food Science and Nutrition, University of the Aegean, 81400 Lemnos, Greece; cgiaginis@aegean.gr; 4 Nutrition and Dietetics Department, University General Hospital of Patras, 26504 Patras, Greece; ioannapartsalaki@gmail.com; 5 Faculty of Medicine, University of Thessaly, 41110 Larissa, Greece; b.mpismpardi@gmail.com (V.B.); imigdanis@uth.gr (I.M.); 6 Department of Clinical Nutrition, Oncology Clinic and Hemodialysis Unit, “E. Patsidis” Health Group, 41335 Larissa, Greece; 7 Department of Nutritional Sciences and Dietetics, International Hellenic University, 57001 Thessaloniki, Greece; agpritsa@ihu.gr; 8 Department of Nephrology, General Hospital of Larissa, 41221 Larissa, Greece; polyzoukonsta@yahoo.com

**Keywords:** blenderized tube feeds, commercial enteral formulas, gastrointestinal symptoms, stool characteristics, enteral feeding complications, clinical nutrition

## Abstract

*Background and Objectives*: Accumulating evidence suggests that blenderized tube feeds (BTFs) may be generally well tolerated from a gastrointestinal (GI) perspective. Previous studies, predominantly conducted in pediatric populations, have reported improvements in symptoms such as gagging, retching, emesis, diarrhea, and vomiting following the transition from commercial enteral formulas (CEFs) to BTFs. However, data in adult populations remain limited. The primary objective of the present study was to investigate the association between different types of enteral feeds and gastrointestinal tolerance in adult patients receiving enteral nutrition. *Materials and Methods*: A prospective observational cohort study design was employed. Data were collected using a structured questionnaire, assessing parameters including recent medical history, weight changes, anthropometric characteristics, biochemical markers, gastrointestinal symptoms, and stool characteristics at baseline and after 30 days of enteral nutrition (EN). Participants (*n* = 60) were stratified into two groups: (i) the CEF group, receiving exclusively commercial enteral formulas, and (ii) the CEF–BTF group, receiving a combination of commercial formulas and blenderized tube feeds. *Results*: The mean body mass index (BMI) of the participants was within the normal range (21.7 ± 3.9 kg/m^2^). The prespecified primary outcome (total GSRS score at 30 days) did not differ significantly between groups. At 30 days following EN initiation, the CEF group reported significantly higher median discomfort scores for diarrhea [4 (1–5)] and loose stools [4 (1–4)] compared with the CEF–BTF group [1 (1–2)] and [1 (1–1.5)], respectively (r = 0.396; *p* = 0.016 and r = 0.485; *p* = 0.016). Additionally, stool consistency differed significantly between groups. Participants in the CEF group were more frequently classified as having loose stools, and participants in the CEF-BTF group were more frequently classified as having normal or hard stools (Cramer’s V = 0.493; *p* = 0.003). *Conclusions*: In conclusion, the findings of the present study suggest that the inclusion of BTFs may be associated with improvements in specific gastrointestinal outcomes compared with commercial enteral formulas alone in adult patients receiving enteral nutrition. Nevertheless, the current body of evidence remains heterogeneous and inconclusive, likely reflecting the multifactorial nature of gastrointestinal symptoms in this population. Further research, particularly well-designed randomized controlled trials in adult cohorts, is warranted to elucidate the impact of enteral feeding modalities on gastrointestinal outcomes.

## 1. Introduction

Enteral nutrition (EN) refers to the delivery of nutrients directly into the gastrointestinal tract via a feeding tube in patients who are unable to meet their nutritional requirements orally but have a functioning gut [1,2]. It is commonly used in individuals with neurological conditions affecting swallowing, such as stroke and motor neuron disease, as well as in patients with head and neck cancers or structural abnormalities that impair oral intake [3]. It is also frequently required in critically ill patients, particularly those in intensive care units or receiving mechanical ventilation [4]. In addition, enteral nutrition may be indicated in patients with severe disease-related malnutrition or markedly reduced oral intake, where nutritional requirements cannot be achieved through normal feeding [2,5]. Overall, enteral feeding is considered when oral intake is unsafe, insufficient or not feasible, but gastrointestinal function is preserved. Compared with parenteral nutrition, enteral feeding is generally preferred due to its lower risk of infectious complications and its role in maintaining gut integrity and immune function. Moreover, early initiation of enteral nutrition has been associated with improved clinical outcomes, including reduced length of hospital stay and decreased morbidity in certain patient populations [3].

Enteral feeds are broadly classified into commercial enteral formulas (CEFs) and blenderized tube feeds (BTFs). Commercial formulas are liquid nutritional preparations designed for tube feeding, typically providing macronutrients from sources such as milk or soy proteins, carbohydrate components like maltodextrin and fats derived from vegetable oils [3]. These formulas are further categorized into standard (polymeric), semi-elemental or elemental, immunomodulating and disease-specific formulations. They are formulated to provide complete and balanced nutrition, ensuring precise nutrient delivery and ease of administration in clinical settings. However, concerns have been raised regarding their lack of dietary variety and potential impact on gut microbiota composition [3].

Blenderized feeding, on the other hand, refers to the administration of pureed or liquified foods through an enteral feeding device for individuals who are unable to achieve adequate nutrition by mouth. Blenderized feeds are composed of whole, natural ingredients and can either be commercially prepared or homemade [6]. They are suitable for use in both hospital and home settings, though they come with both benefits and potential drawbacks. Both types of feeds are delivered directly to the gastrointestinal tract via a tube, catheter or stoma. Despite increasing interest in blenderized feeding, challenges such as variability in nutrient composition, risk of microbial contamination, and tube clogging must be carefully considered in clinical practice [7].

Accumulating research suggests that BTFs may be associated with satisfactory GI function and tolerance. Several studies conducted in pediatric enterally fed patients have shown improvement in symptoms such as gagging, retching and emesis when transitioning from commercial enteral formulas to blenderized feeds containing whole, natural foods [8,9,10]. Moreover, other research has reported a significant reduction in the frequency of watery stool evacuations and diarrhea [11] as well as improvements in other GI symptoms such as vomiting, nausea and abdominal pain [12] among patients receiving blended diets. Potential explanations include the higher viscosity of blended diets, differences in fiber composition between the two types of feeds and the presence of phytonutrients and natural probiotics in whole foods. Additionally, blenderized diets may promote greater microbial diversity within the gut, which has been associated with improved gastrointestinal health and function. Although existing research has detected some beneficial effects of BTFs, the evidence remains limited, heterogeneous and predominantly focused on pediatric populations. Therefore, the primary aim of the present study is to provide some insight into the association between different types of enteral feeds (blenderized and commercial) and gastrointestinal tolerance in adult patients receiving enteral nutrition.

## 2. Materials and Methods

This was a multicenter, prospective observational cohort study. The study was initiated in September 2025 and completed in March 2026. The study was conducted among 60 male and female adult patients receiving enteral nutrition via nasogastric or gastrostomy tube. Using an administered questionnaire, several parameters were assessed and recorded, including recent medical and weight history, anthropometric characteristics, biochemical markers and gastrointestinal symptoms, before enteral feeding initiation (baseline) and after 30 days on enteral feeding (follow-up). Data collection was carried out in several departments (internal medicine, neurology, otolaryngology and oncology) at University General Hospital of Larissa, University General Hospital of Patras and General Hospital of Patras. The questionnaires were administered both in printed form and electronically. A registered dietitian was available during questionnaire administration to provide clarification when needed. Participants with an acute or chronic condition or exposures (e.g., active infection, food allergies, active inflammatory bowel disease, irritable bowel syndrome, undergoing chemo/radiotherapy, receiving antibiotics, recent surgery of the stomach or lower gastrointestinal tract) that might have affected their nutritional intake or their gastrointestinal function were excluded from the study. As specified before patient enrollment, patients who required antibiotic therapy during the follow-up period were excluded from the final analysis. The need for antibiotic treatment was anticipated in this hospitalized population, and antibiotics have known effects on GI symptoms. Furthermore, patients who were unable to communicate with the researchers collecting the data due to their medical condition were also excluded from the study. Among patients eligible for inclusion (*n* = 85), 15 (9 males and 6 females) declined to participate in the study. Additionally, 10 patients were excluded due to initiation of antibiotic therapy during the 30-day follow-up period. Of the 10 patients excluded for this reason, six were in the CEF group and four in the CEF-BTF group. A formal sample size calculation was not performed a priori due to the exploratory and observational design of the study. The sample size was determined based on the number of eligible patients receiving enteral nutrition during the study period at the participating hospitals. The final sample size is comparable to previous studies evaluating GI tolerance in enterally fed patients, which is relatively small due to strict inclusion criteria and clinical recruitment constraints.

The Gastrointestinal Symptom Rating Scale (GSRS) was used to assess participants’ gastrointestinal symptoms. Specifically, the official Greek-language version of the tool was used, provided by the copyright holder (AstraZeneca AB, Mölndal, Sweden). The GSRS is a widely recognized and validated tool for assessing gastrointestinal symptoms in clinical and research settings [13] and has previously been used in studies involving patients with neurological disorders [14,15]. The GSRS consists of 15 questions and covers five key domains (reflux, abdominal pain, indigestion, diarrhea and constipation). Each of the 15 questions addresses a GI symptom and is rated on a 7-point Likert scale where higher scores indicate more severe symptoms (1 = no discomfort, 7 = very severe). To assess stool characteristics of the participants the Bristol Stool Chart [16] was used which is a validated medical tool designed to classify the shape and consistency of human feces into 7 distinct categories (types 1 and 2 indicating constipation, types 3 and 4 being the most ideal and types 5, 6 and 7 indicating diarrhea). The tool was developed at the Bristol Royal Infirmary as a clinical assessment tool in 1997 and has since been widely used in both clinical and experimental fields. Regarding questionnaire completion, questionnaires were completed by the participants whenever possible. When physical limitations prevented self-completion, a caregiver assisted by reading the questions and recording the participant’s responses. Proxy responses were not permitted. Patients unable to communicate their own responses were excluded. Several biochemical markers, including serum albumin (g/dL), potassium (mmol/L), sodium (mmol/L), phosphorus (mg/dL) and glucose (mg/dL) were also measured and quantified with an automatic biochemical analyzer in all participants. A small number of baseline and follow up biochemical measurements were unavailable because certain laboratory reagents were temporarily unavailable at the hospital laboratory. Analyses of biochemical outcomes were therefore performed using the available data for each parameter. The prespecified primary outcome was the total GSRS score at 30 days. Secondary outcomes included the individual GSRS symptom scores, Bristol stool chart, body weight, bowel frequency and biochemical parameters. Informed consent was obtained from all participants prior to study enrollment. The study protocol was approved by the Ethics Committee of the University of Thessaly (ethics approval code number 55/25-07-2025), and was conducted in accordance with the Helsinki declaration.

### 2.1. Groups of the Study and Received Feeds

A consecutive sample of eligible patients requiring enteral nutrition was recruited. Enteral nutrition was initiated at study enrollment and participants were prospectively followed for 30 days. The participants of the study were classified into two groups: the CEF group, which received exclusively commercial enteral formulas to meet their nutritional requirements, and the CEF-BTF group, which received a combination of commercial formulas and blenderized tube feeds. The formulas used in the CEF group were polymeric standard formulas (per 100 mL: energy 101 kcal, protein 4 g, carbohydrates 13.6 g, fat 3.4 g, fiber 0 g) as well as semi-elemental formulas with different nutritional composition (energy 150 kcal, protein 6.75 g, carbohydrates 18.4 g, fat 5.5 g, fiber 0 g; or energy 100 kcal, protein 4 g, carbohydrates 12.8 g, fat 4 g, fiber 0 g). Participants in the CEF-BTF group received the same types of commercial formulas in combination with blenderized feeds, primarily consisting of chicken or beef, rice or pasta, carrots, other vegetables (courgettes) and yogurt. The typical weekly pattern consisted of chicken and rice or pasta approximately four times per week and beef with pasta approximately three times per week, with vegetables and yogurt included in both meal types. All patients were fed via bolus administration for at least one month. In the CEF-BTF group one of the daily bolus meals was a blenderized feed. The nutritional composition of the blenderized feed was estimated using a nutritional analysis software (SOMA Nutri, Serinth, Greece) that included the United States department of agriculture (USDA) food composition database. The feed provided approximately 700 kcal, 40 g of protein, 80 g of carbohydrate, 25 g of fat and 8 g of fiber, with some variability depending on ingredient selection. Formula selection was based on individual clinical and nutritional requirements, and the authors of the study had no involvement in determining the feeding regimen of the participants.

### 2.2. Statistical Analysis

Statistical analysis was conducted using Statistical Product and Service Solutions, version 26 (IBM SPSS Statistics for Windows, Armonk, NY, USA). The Kolmogorov–Smirnov test was carried out to determine the normality of the distribution of the examined variables. Continuous variables, such as anthropometric characteristics, are presented as means ± standard deviations. For baseline characteristics and follow-up clinical and biochemical outcomes continuous variables were compared using the independent samples *t*-test (for normally distributed data) or the Mann–Whitney U test (for not normally distributed data) and categorical variables were compared using Pearson’s chi-square test. To assess possible differences in gastrointestinal symptom severity between the commercial enteral formula group and the group that received a combination of commercial formulas and blenderized feeds, the Mann–Whitney U test was used. To account for multiple comparisons across the GI symptom outcomes, Holm-Bonferroni adjusted *p*-values were calculated across the individual 15 GSRS symptom scores and the ordinal BSC score. No multiplicity adjustment was applied to the prespecified primary outcome. To account for potential residual confounding, analysis of covariance (ANCOVA) was performed. Statistical significance was maintained at *p* < 0.05.

## 3. Results

A total of 60 patients were included in the final analysis. The mean age of the participants was 59.3 ± 16.2 years (61.7% males and 38.3% females). The participants’ mean BMI was within the normal range (21.7 ± 3.9 kg/m^2^). Neurodegenerative diseases were the most common indication for artificial/enteral nutrition. No significant differences were detected in sex, age, diagnosis, anthropometric characteristics, GSRS and biochemical indices between the two study groups at baseline (Table 1).

The prespecified primary outcome (total GSRS score) did not differ significantly between groups. Regarding the secondary outcomes related to gastrointestinal symptoms, no significant differences were observed concerning the first three domains of the assessment tool (reflux, abdominal pain and indigestion) between the CEF group and the BTF-CEF group one month after initiation of enteral feeding. As for diarrhea and loose stools, the CEF group reported significantly higher median discomfort scores [4 (1–5)] and [4 (1–4)] compared to the BTF-CEF group [1 (1–2)] and [1 (1–1.5)], respectively, at 30 days after enteral feeding initiation (r = 0.396; *p* = 0.016 and r = 0.485; *p* = 0.016 respectively) (Table 2). The CEF group reported significantly lower median GSRS hard stool symptom and sensation of incomplete bowel evacuation scores than the CEF-BTF group at 30 days; however, these differences were no longer significant after Holm-Bonferroni correction (*p* = 0.276 and *p* = 0.528, respectively). Furthermore, the distribution of BSC categories differed significantly between groups at 30 days after feeding initiation, with participants in the CEF group more frequently classified as having loose stools and participants in the CEF-BTF group more frequently classified as having normal or hard stools (Cramer’s V = 0.493; *p* = 0.003) (Table 2). Concerning the biochemical markers measured, no statistical differences were observed in any of them at 1 month on EN between the two groups. Furthermore, no significant differences in bowel movement frequency were recorded after 1 month of enteral feeding between the two groups under investigation, although the CEF group exhibited higher mean values (Table S1). Lastly, Figure 1 summarizes the significant differences in GI symptoms and stool characteristics between the two groups of the study. To account for potential confounding, additional analysis was performed using ANCOVA (sensitivity analysis) adjusting for the baseline value for each GI symptom, BMI and age. The adjusted results were consistent with the initial findings (Table 3).

## 4. Discussion

The findings of the present study suggest that, although overall gastrointestinal symptom burden did not differ significantly between the two groups, patients receiving commercial enteral formulas (CEFs) presented higher gastrointestinal symptom scores for diarrhea and loose stools, as well as higher BSC scores (indicating looser stool consistency), than those receiving a combination of blenderized tube feeds and commercial formulas (CEF-BTF). These results are consistent with the existing body of literature, which, although limited and predominantly focused on pediatric populations, indicates that blenderized feeding is generally associated with improved gastrointestinal tolerance [8,9,10,11]. One of the few studies conducted in adult populations reported comparable findings [11]. This randomized controlled trial, conducted in a rehabilitation center in Germany, included 118 patients recovering from neurological conditions such as ischemic stroke or intracranial hemorrhage who required enteral nutrition. Participants were randomly assigned to receive either blenderized feeding or a commercial formula for up to 30 days. Outcomes including bowel movement frequency and stool consistency, assessed using the Bristol Stool Chart, demonstrated that patients receiving natural, blenderized feeds experienced a significant reduction in watery stools and episodes of diarrhea compared with those receiving standard enteral formulas. These improvements may be attributable to differences in fiber composition, as well as the presence of bioactive components in whole foods—such as pectins and acidic oligosaccharides derived from carrots—which have been shown to exert beneficial gastrointestinal effects [17,18].

Evidence from multiple meta-analyses further supports the role of dietary fiber in improving gastrointestinal outcomes, suggesting that high-fiber enteral feeding may reduce diarrhea in non-critically ill patients [19,20,21]. A recent study conducted in Saudi Arabia involving tube-fed adult patients reported that fiber-enriched enteral formulas significantly reduced the incidence of diarrhea by 46% and weight loss by 41% [22]. Dietary fiber, composed primarily of non-digestible carbohydrates, undergoes fermentation by the intestinal microbiota, producing gases and short-chain fatty acids (SCFAs), including acetate, propionate, and butyrate. These metabolites serve as key energy substrates for colonocytes and play a critical role in epithelial regeneration, enhancement of fluid and electrolyte absorption, regulation of intestinal motility, maintenance of barrier integrity, and reduction in bacterial translocation [23,24,25]. Notably, soluble and fermentable fibers—such as pectin and inulin—are more readily metabolized by gut microbiota and are commonly found in plant-based foods, including grains, vegetables, and legumes [26]. In the present study, participants in the CEF group consumed substantially lower amounts of soluble fiber, as many received standard polymeric or elemental formulas that typically contain little or no fiber.

Despite these findings, the literature remains inconclusive regarding the efficacy of fiber-enriched formulas in reducing diarrhea among enterally fed patients. A systematic review by del Olmo et al., which included 25 studies, reported no significant difference in bowel movement frequency between fiber-containing and fiber-free formulas in patients with normal gastrointestinal function [27]. However, subgroup analyses suggested that fermentable fiber may reduce the incidence of diarrhea in critically ill and postoperative populations, although the overall quality of evidence remains limited. Similarly, several randomized controlled trials have failed to demonstrate a significant reduction in diarrhea when comparing fiber-enriched with fiber-free formulas [28,29]. The heterogeneity observed across studies may be attributed to methodological limitations, including small sample sizes, inconsistent definitions of diarrhea, lack of differentiation between soluble and insoluble fiber types, and the presence of confounding factors such as antibiotic use, infections (e.g., C. difficile), and concomitant medications. Future research should prioritize well-designed randomized controlled trials with standardized outcome measures, clear characterization of fiber types, homogeneous patient populations, and appropriate control of confounders. Regarding carrot-derived pectins and acidic oligosaccharides, pectin is a highly fermentable soluble fiber, while acidic oligosaccharides may act as prebiotics by promoting the growth of beneficial bacteria such as Bifidobacteria [30,31,32]. In the present study, the CEF-BTF group consumed carrots daily, as they were included in the blenderized feeds administered.

In addition to fiber content, the improved gastrointestinal tolerance associated with blenderized diets may be partially explained by their increased viscosity. Evidence from pediatric populations, including children undergoing Nissen fundoplication or those with neurological impairments, indicates that transitioning from commercial formulas to blenderized diets is associated with reductions in symptoms such as retching, gagging, diarrhea, and emesis [8,10]. The higher viscosity of blenderized feeds is thought to slow gastric emptying, thereby reducing the risk of dumping syndrome. Dumping syndrome commonly occurs following gastric surgery or in conditions associated with impaired gastric motility, where rapid gastric emptying leads to the delivery of hyperosmolar contents into the small intestine [33,34,35,36,37]. This results in fluid shifts from the intravascular compartment into the intestinal lumen, causing distention and symptoms such as abdominal pain, nausea, bloating, and diarrhea [38,39]. In contrast, whole or minimally processed foods may undergo more gradual digestion, allowing for controlled release of chyme into the small intestine and promoting a more physiological hormonal response involving mediators such as glucagon-like peptide-1, insulin, cholecystokinin, and bradykinin. This may contribute to improved gastrointestinal motility and symptom control. Furthermore, the gradual digestion of complex polysaccharides in blenderized diets may support more stable glycemic responses [8]. The participants in the CEF group mainly received either standard polymeric or semi-elemental/elemental formulas, which are typically less viscous since they are engineered to be flowable and pass easily through feeding tubes [6]. However, the viscosity and osmolarity of the administered feeds were not directly measured in the present study. Therefore, although these physicochemical properties represent plausible mechanisms through which blenderized feeds may influence gastrointestinal tolerance, their contribution to the observed differences between groups cannot be established from the present data.

Another potential mechanism underlying the observed benefits of blenderized feeding is its influence on the gut microbiota. Emerging evidence suggests that blenderized diets are associated with increased microbial diversity and a more favorable microbial profile [7]. The diverse composition of blenderized feeds—including dietary fiber, phytonutrients, and naturally occurring prebiotic compounds—may support a more complex and resilient microbial ecosystem [40,41,42]. Several studies in pediatric populations have demonstrated that transitioning from commercial formulas to blenderized diets is associated with increased bacterial richness and diversity, as well as shifts toward microbial communities linked to improved gastrointestinal health [9,43,44]. These effects have been attributed to the inclusion of whole foods rich in fermentable substrates that promote short-chain fatty acid production.

However, findings across studies are not entirely consistent. Some investigations have reported no significant differences in gastrointestinal symptoms or microbiome composition between blenderized and commercial feeding groups [45]. Moreover, current understanding of the role of the gut microbiota in disease remains incomplete, although reduced microbial diversity has been associated with a range of adverse health outcomes [46,47]. Importantly, the majority of existing studies have been conducted in pediatric populations, limiting the generalizability of findings to adults. Consequently, further large-scale, well-controlled studies focusing on adult populations are warranted.

The present study has several limitations that should be acknowledged. The relatively small sample size and observational design limit the generalizability of the findings and preclude causal inferences. Additionally, the assessment of gastrointestinal symptoms using the Gastrointestinal Symptom Rating Scale (GSRS) is inherently subjective and may be influenced by recall bias and inter-individual variability in symptom perception. The nutritional composition of the blenderized feeds was estimated using nutritional analysis software based on standard food-composition data rather than determined by direct laboratory analysis of individual prepared meals. Although this approach enabled estimation of nutrient composition under routine clinical feeding conditions, variability in ingredient selection and preparation may have resulted in small differences between the estimated and actual nutrient composition of individual feeds. Furthermore, physicochemical characteristics of the prepared feeds, including osmolarity and viscosity, were not directly measured; therefore, their potential contribution to the observed gastrointestinal outcomes could not be determined. Another consideration relates to the possibility of microbial contamination. Microbiological analyses of the administered feeds were not performed. Although both groups received enteral nutrition by bolus administration, the preparation of the blenderized meals involved additional food preparation and handling, which may introduce opportunities for microbial contamination. Therefore, contamination and its potential influence on gastrointestinal outcomes could not be directly assessed. Nevertheless, the findings contribute to the growing body of evidence supporting the potential benefits of blenderized feeding and provide a foundation for future research involving larger cohorts and controlled interventional designs.

## 5. Conclusions

In conclusion, although the prespecified outcome (total GSRS score) did not differ significantly between groups, the findings of the present study suggest that the inclusion of BTFs may be associated with improvements in specific gastrointestinal outcomes compared with commercial enteral formulas alone in adult patients receiving enteral nutrition. Compared with commercial formulas alone, patients receiving additional BTFs experienced less diarrhea and fewer loose stools, together with improved stool consistency. Although the existing literature generally supports an association between BTFs and reduced gastrointestinal symptoms, definitive conclusions remain challenging due to the heterogeneity and inconsistency of available evidence. This variability is likely multifactorial, reflecting the complex etiology of gastrointestinal symptoms in enterally fed patients, which extends beyond dietary factors to include influences such as antibiotic use, infections, and underlying disease states. Additionally, variations in feeding practices—particularly differences in administration methods (e.g., bolus versus continuous feeding) and infusion rates—may further contribute to discrepancies in reported outcomes.

Moreover, the lack of standardization in the definition and composition of BTFs, including differences between homemade and commercially prepared formulations and their respective macronutrient profiles, represents an additional source of variability across studies. Consequently, further research is warranted, particularly well-designed randomized controlled trials in adult populations, to more clearly elucidate the impact of different enteral feeding modalities on gastrointestinal tolerance and related outcomes.

In clinical practice, the selection of an appropriate enteral feeding regimen remains complex and should be individualized based on a comprehensive assessment of patient-specific factors. These include the underlying clinical condition, metabolic demands, gastrointestinal function, and specific nutritional requirements. Consideration should also be given to the compositional characteristics of the feed, including macronutrient distribution, fiber content, and osmolarity, as well as the route and method of administration. Furthermore, safety considerations—such as the risk of feeding intolerance and microbial contamination—alongside practical aspects, including availability, cost, and feasibility in both hospital and home settings, are essential in guiding optimal nutritional management.

## Figures and Tables

**Figure 1:**
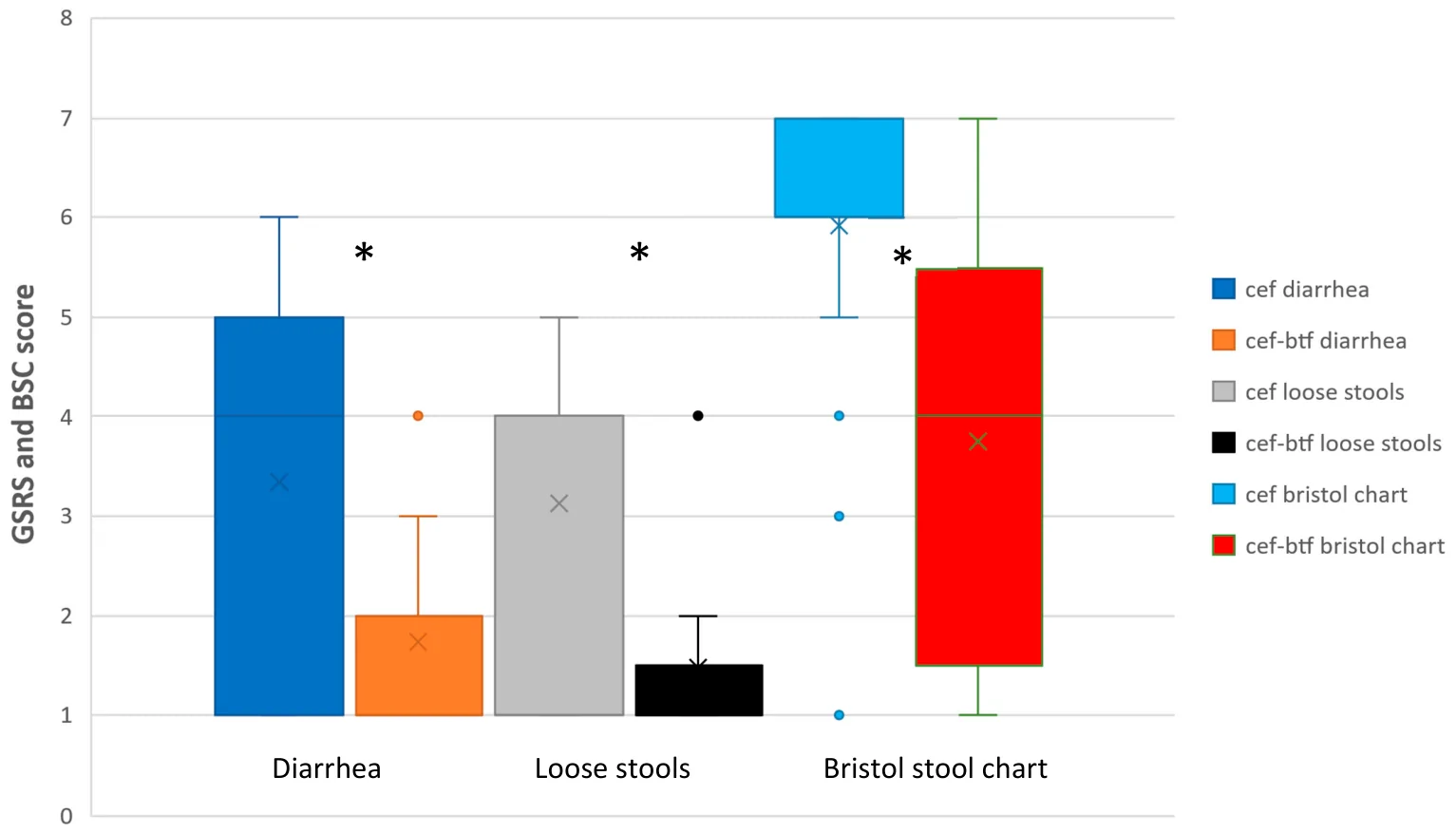
Box-plot showing the significant differences in GI symptoms between CEF and CEF-BTF groups (GSRS and BSC), 1 month after EN initiation. * indicates significant between-group differences in symptom severity (diarrhea, loose stools) and bristol stool chart scores.

**Table 1:** Demographic, anthropometric and biochemical measured parameters of the participants at baseline.

Variables	Commercial Formula (Mean ± SD/Number of Cases) *n* = 31	Blenderized Feed and Commercial Formula (Mean ± SD/Number of Cases) *n* = 29	*p* Value
Gender			0.838
Males	20	17	
Females	11	12	
Diagnosis			0.653
Neurodegenerative diseases	18	15	
Stroke	7	9	
Cancer	5	3	
Other	1	2	
Age (years)	56 ± 18	62 ± 13	0.204
Weight (kg)	62.5 ± 14	63.5 ± 16	0.833
BMI (kg/m^2^)	21.3 ± 3.4	22.2 ± 4.3	0.301
Serum Albumin (g/dL)	3.6 ± 0.6	3.6 ± 0.4	0.859
Sodium (mmol/L)	140.2 ± 3.9	139 ± 4	0.524
Potassium (mmol/L)	4.1 ± 0.84	4.4 ± 0.73	0.173
Serum phosphorus (mg/dL)	3 ± 1	3.2 ± 0.6	0.696
Serum glucose (mg/dL)	117.4 ± 46	118.2 ± 27	0.966
GSRS	25.6 ± 6.4	24.9 ± 5.3	0.784

BMI, body mass index; SD, standard deviation; GSRS, gastrointestinal symptom rating scale.

**Table 2:** Gastrointestinal symptom severity between CEF and CEF-BTF groups 1 month after enteral feeding initiation.

**GSRS Symptom Scale** **-** **No discomfort** **Minor discomfort ** **Mild ** **Moderate** **Moderately severe** **Severe** **Very severe**	**Commercial Enteral Formula Group,** **Mean ± SD/Median (IQR)**	**Blenderized Feed—Commercial Enteral Formula Group, Mean ± SD/Median (IQR)**	**Effect Size (r)/mean Difference (95% CI)**	***p*** ** Value**	**Holm-Adjusted ** ***p***
**Primary outcome**					
Total GSRS score	26.2 ± 6.2	25.1 ± 6.3	1.05 (−2.18 to 4.29)	0.521	-
**Secondary outcomes—GSRS symptoms**					
Pain upper abdomen	1 (1–1)	1 (1–1)	r = 0.118	0.361	1.000
Heartburn	1 (1–1)	1 (1–2)	r = 0.128	0.322	1.000
Acid reflux	1 (1–1)	1 (1–1)	r = 0.004	0.973	1.000
Hunger pains	1 (1–2)	1 (1–1)	r = 0.204	0.179	1.000
Nausea	1 (1–1)	1 (1–1)	r = 0.046	0.720	1.000
Rumbling	2 (1–2.5)	2 (1–2)	r = 0.069	0.480	1.000
Bloating	1 (1–2)	1 (1–1.5)	r = 0.059	0.545	1.000
Belching	1 (1–2)	1 (1–2.5)	r = 0.066	0.767	1.000
Breaking wind	2 (2–3)	2 (2–4)	r = 0.049	0.704	1.000
Constipation	1 (1–2)	2 (1–4)	r = 0.172	0.183	1.000
Diarrhea	4 (1–5)	1 (1–2)	r = 0.396	0.001	**0.016**
Loose stools	4 (1–4)	1 (1–1.5)	r = 0.485	0.001	**0.016**
Hard stools	1 (1–1)	1.5 (1–3)	r = 0.310	0.023	0.276
Urgent need to have a bowel movement	1 (1–2)	1 (1–1)	r = 0.192	0.137	1.000
Sensation of not completely emptying the bowels	1 (1–1)	1.5 (1–2)	r = 0.298	0.048	0.528
**Secondary outcomes—stool characteristics**					
Bristol stool chart	6 (6–7)	4 (1.5–5.25)	r = 0.442	0.001	**0.016**
**Bristol stool chart categories**					
	Hard 1 (3.2%) Normal 3 (9.7%) Loose 27 (87%)	Hard 10 (34.5%) Normal 7 (24.1%) Loose 12 (41.4%)	Cramer’s V = 0.493	**0.003**	-

IQR, interquartile range; GSRS, gastrointestinal symptom rating scale; SD, standard deviation. Statistically significant *p*-values are shown in bold.

**Table 3:** ANCOVA-adjusted comparison of gastrointestinal symptom severity between CEF and CEF-BTF groups at 30 days.

**GSRS Symptom Scale** **-** **No discomfort** **Minor discomfort** **Mild** **Moderate** **Moderately severe** **Severe** **Very severe**	**Commercial Enteral Formula Group, Adjusted Mean**	**Blenderized Feed—Commercial Enteral Formula Group, Adjusted Mean**	**Mean Difference (95% CI)**	***p*** ** Value**
Pain upper abdomen	1 (0.15)	1.4 (0.14)	−0.41 (−0.84 to 0.01)	0.083
Heartburn	1.2 (0.19)	1.6 (0.18)	−0.36 (−0.91 to 0.18)	0.186
Acid reflux	1.25 (0.15)	1.39 (0.14)	−0.13 (−0.56 to 0.29)	0.521
Hunger pains	1.6 (0.20)	1.3 (0.18)	0.28 (−0.28 to 0.84)	0.320
Nausea	1.17 (0.08)	1.16 (0.07)	0.009 (−0.22 to 0.24)	0.935
Rumbling	1.8 (0.17)	1.8 (0.15)	−0.069 (−0.53 to 0.40)	0.767
Bloating	1.4 (0.20)	1.3 (0.18)	0.078 (−0.47 to 0.63)	0.777
Belching	1.5 (0.17)	1.8 (0.15)	−0.29 (−0.76 to 0.18)	0.224
Breaking wind	2.3 (0.22)	2.5 (0.19)	−0.16 (−0.76 to 0.44)	0.598
Constipation	1.7 (0.30)	2.5 (0.28)	−0.83 (−1.6 to 0.13)	0.059
Diarrhea	3.1 (0.19)	2 (0.18)	1.100 (0.54 to 1.66)	**0.001**
Loose stools	2.9 (0.18)	1.7 (0.17)	1.22 (0.69 to 1.75)	**0.001**
Hard stools	1.4 (0.14)	1.8 (0.13)	−0.34 (−0.75 to 0.07)	0.102
Urgent need to have a bowel movement	1.4 (0.14)	1.1 (0.12)	0.29 (−0.09 to 0.69)	0.134
Sensation of not completely emptying the bowels	1.5 (0.10)	1.6 (0.08)	−0.13 (−0.40 to 0.13)	0.316
Bristol stool chart	5.5 (0.21)	4 (0.20)	1.47 (0.86 to 2.08)	**0.001**

Values are estimated marginal means (standard errors) derived from ANCOVA, adjusting for the baseline value for each GI symptom, age and BMI. Statistically significant *p*-values are shown in bold.

## Data Availability

The data generated and analysed for this study are available from the corresponding author upon reasonable request.

## Supplementary Materials

The following supporting information can be downloaded at: https://www.mdpi.com/article/10.3390/medicina62101839/s1. Table S1: Follow up clinical and biochemical outcomes at 30 days.
