# Supplementary material for: Effects of Adding Blenderized Tube Feeds to Commercial Enteral Formulas on Gastrointestinal Symptoms in Adult Patients Receiving Enteral Nutrition
Source: Medicina (Kaunas). 2026 Sep 23;62(10):1839. doi: 10.3390/medicina62101839 (PMC13707139; doi:10.3390/medicina62101839)
Supplement: Supplementary file 1 [file medicina-62-01839-s001.zip › medicina-4490947-supplementary.pdf]

Supplementary table S1. Follow up clinical and biochemical outcomes at 30 days

Variables	Commercial Formula (mean ± SD) n=31	Blenderized feed and Commercial formula (mean ± SD) n=29	P value
Weight (kg)	61.2±13.9	62.3±15.7	0.776
Serum Albumin (g/dl)	3.8±0.6	3.9±0.3	0.901
Sodium (mmol/L)	140±3.7	139.5±3.4	0.783
Potassium (mmol/L)	4.2±0.4	4.4±0.3	0.461
Serum phosphorus (mg/dl)	3.1±0.6	3.5±0.4	0.212
Serum glucose (mg/dl)	119.4±38	115.2±13	0.762
Bowel movement frequency/week	10.1±6.8	7.6±7	0.222

SD, standard deviation
